# Case report: thigh hidradenoma masquerading as lipoma

**DOI:** 10.1093/jscr/rjae518

**Published:** 2024-08-24

**Authors:** Abdullah Alhaqbani, Homoud Alawfi, Firas Alahmadatea, Sami Almalki

**Affiliations:** General Surgery Department, King Abdulaziz Medical City, Riyadh, Saudi Arabia; General Surgery Department, King Abdulaziz Medical City, Riyadh, Saudi Arabia; General Surgery Department, King Abdulaziz Medical City, Riyadh, Saudi Arabia; General Surgery Department, King Abdulaziz Medical City, Riyadh, Saudi Arabia

**Keywords:** general surgery, lipoma, hidradenoma

## Abstract

Hidradenomas are benign adnexal neoplasms, which were recently been subdivided into two groups: eccrine differentiation (poroid hidradenomas) or apocrine differentiation (clear cell hidradenomas) with the latter being rarer. These types of tumors have been associated with recurrence and malignant transformation; however, recurrence and malignancy are considered very rare. We present a case report of a 35-year-old male who presented with two lumps, clinically representing simple lipomas but one of them turned to be a hidradenoma. A 35-year-old gentleman not known to have any medical illnesses and surgically free, presented to our general surgery clinic complaining of two slow-growing (over 3 years) painless lumps, one in the right upper thigh and the other one in the left shoulder. The patient denied any previous history of trauma or infection nor any history of discharge or overlying skin changes, and there were no clinical features that might suggest the presence of malignancy. Upon examination, both lumps were firm, freely mobile, non-tender, intact overlying skin, with no skin changes, and no regional lymphadenopathy. Prior to excision, our preliminary impression was lipoma for both masses. Surgical excision was carried out with clear margins; each mass was labeled separately, and specimens were sent for histopathology. Histopathological diagnosis of the left shoulder mass was consistent with lipoma; however, the right upper thigh mass turned to be a hidradenoma. Hidradenomas are uncommon benign neoplasms with varied types. Recurrence and transformation into malignancy have been reported in some cases. Complete surgical excision with negative margins and further follow-up with the patient are crucial to prevent such consequences. Clinical diagnosis can be difficult; however, the management is the same with surgical removal as it will give us the definitive diagnosis with the pathology report.

## Introduction

Hidradenomas are benign adnexal neoplasms, which were recently been subdivided into two groups: eccrine differentiation (poroid hidradenomas) or apocrine differentiation (clear cell hidradenomas) with the latter being rarer [1]. These tumors are considered relatively rare, and they are commonly found in adults, rarely in children, with most cases affecting females more than males [2].

The affected region is usually head and neck, trunk, and extremities, presenting as a firm dermal nodule spanning between 3 and 30 mm in size, and they grow slowly with occasional overlying skin changes [2,3]. These types of tumors have been associated with recurrence and malignant transformation; however, recurrence and malignancy are considered very rare [3].

We present a case report of a 35-year-old male who presented with two lumps, clinically representing simple lipomas but one of them turned to be a hidradenoma.

## Case report

A 35-year-old gentleman not known to have any medical illnesses and surgically free, presented to our general surgery clinic complaining of two slow-growing (over 3 years) painless lumps, one in the right upper thigh and the other one in the left shoulder. The patient denied any previous history of trauma or infection nor any history of discharge or overlying skin changes, and there were no clinical features that might suggest the presence of malignancy.

Upon examination, both lumps were firm, freely mobile, non-tender, intact overlying skin, with no skin changes, and no regional lymphadenopathy. Prior to excision, our preliminary impression was lipoma for both masses. Surgical excision was carried out with clear margins; each mass was labeled separately, and specimens were sent for histopathology.

**Figure 1:**
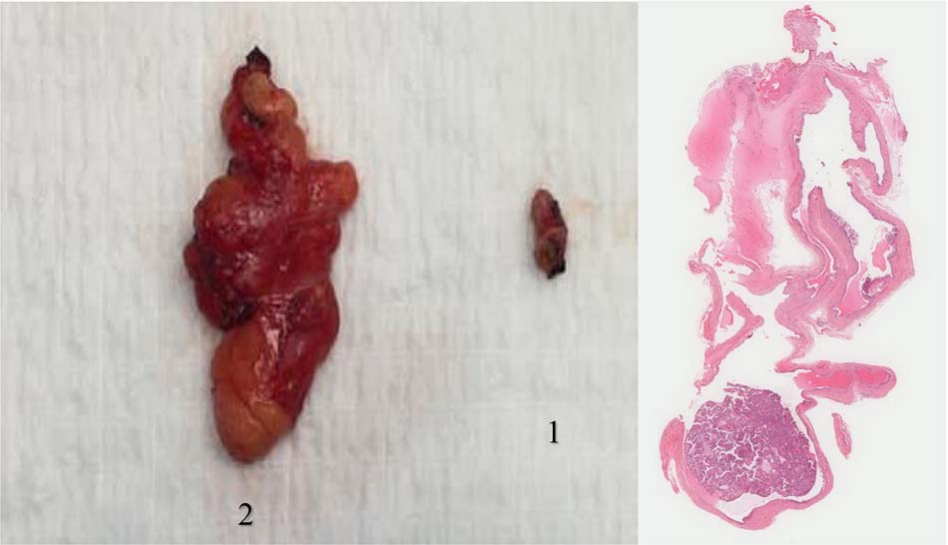
The two masses after surgical excision, along with microscopic slide.

Histopathological diagnosis of the left shoulder mass was consistent with lipoma; however, the right upper thigh mass turned to be a hidradenoma (Fig. 1).

## Pathology report

1: The first specimen represents the upper thigh lesion consisting of brown tan soft tissue measuring 1.4 × 0.9 × 0.5 cm consistent with hidradenoma.

2: The second specimen represents the shoulder lesion on the left side, morphologically demonstrating a homogenous fibrofatty soft tissue measuring 5.6 × 3.4 × 1.5 cm consistent with lipoma.

## Discussion

Hidradenomas are considered a rare benign adnexal neoplasm of uncertain origin [1]. Most traditionally subdivided into either eccrine (more common) or apocrine (rare) derivation [4]. It commonly affect females more than males, and they usually present in head, neck, and extremities with a size range of 3–30 mm [2, 3]. Usually overlying skin is intact upon presentation, but ulceration and discharge (serous) have been described before [4]. The pace of growing is slow; however, rapid growth could represent trauma or malignant changes [4]. Although benign, the malignant transformation has been reported, and it is also associated with recurrence [5, 6]. Tumors that are attached to the skin can sometimes be diagnosed clinically, especially if associated with other symptoms like history or serous discharge, on the other hand, ulcerated lesions may raise the suspicion of basal cell carcinoma [6].

In our case report, hidradenoma presented with a clinical picture of lipoma, especially with two lumps which is common in cases of lipoma to present in any part of the body and multiple lipomas can present at the same time. The management in our case was the same, which was surgical excision, and as a routine of any mass without a prior diagnosis, surgical margins were taken to ensure complete resection. Negative margins were ensured with the histopathology report, and further follow-up was initiated with the patient to ensure elimination of any recurrence.

As there have been reported cases of recurrence of hidradenoma, monitoring and follow-up are necessary to ensure full elimination. The importance of full resection of any mass has been displayed in our case report as without a prior pathology, taking extra precaution is important.

Clinical diagnosis can be difficult in such cases, as the mass was clinically presenting as a lipoma; however, ensuring proper treatment is the key to the management.

## Conclusion

Hidradenomas are uncommon benign neoplasms with varied types. Recurrence and transformation into malignancy have reported in some cases. Complete surgical excision with negative margins and further follow-up with the patient are crucial to prevent such consequences. Clinical diagnosis can be difficult; however, the management is the same with surgical removal as it will give us the definitive diagnosis with the pathology report.
